# Multimodal Imaging of Dual BEST1/EFEMP1-Associated Hereditary Macular Disease

**DOI:** 10.3390/jcm15145495

**Published:** 2026-07-13

**Authors:** Maximilian Pawloff, Marlene Hollaus, Georgios Mylonas, Michael Pircher, Christoph K. Hitzenberger, Mateja Pfeifer, Jose S. Pulido, Graham E. Holder, Stefan Sacu, Markus Ritter

**Affiliations:** 1 Department of Ophthalmology, Medical University of Vienna, Währinger Gürtel 18-20, 1090 Vienna, Austria; maximilian.pawloff@meduniwien.ac.at (M.P.);; 2 Center for Medical Physics and Biomedical Engineering, Medical University of Vienna, Währinger Gürtel 18-20, 1090 Vienna, Austriachristoph.hitzenberger@meduniwien.ac.at (C.K.H.); 3 Institute of Medical Genetics, Medical University of Vienna, Währinger Gürtel 18-20, 1090 Vienna, Austria; 4 Department of Ophthalmology, Wills Eye Hospital, Philadelphia, PA 19107, USA; 5 Department of Ophthalmology, Yong Loo Lin School of Medicine, National University of Singapore, Singapore 117577, Singapore; 6 Department of Ophthalmology, National University Hospital, Singapore 119074, Singapore

**Keywords:** best disease, BD, autosomal dominant drusen, ADD, BEST1, EFEMP1, PS-OCT

## Abstract

**Purpose**: To describe the morphological and functional features of two patients phenotypically and genotypically diagnosed with Best disease and autosomal dominant drusen (BD-ADD) using multimodal imaging. It is hypothesized that the concurrent presence of pathogenic mutations in both BEST1 and EFEMP1 genes is associated with a phenotype that may exhibit characteristic features of both diseases potentially revealing further additive effects on retinal function or structure. The data are compared with those from a patient with genetically confirmed ADD. **Methods**: The patients received a full ophthalmological investigation, including fundus autofluorescence (FAF) imaging, spectral-domain optical coherence tomography (SD-OCT) and polarization-sensitive optical coherence tomography (PS-OCT). Genetic analysis of DNA samples was performed by targeted whole-exome sequencing. **Results**: Macular SD-OCT demonstrated a thickened retinal pigment epithelium (RPE)-Bruch’s membrane complex corresponding to macular drusen-like deposits in both ADD and BD-ADD cases. In contrast to isolated ADD, BD-ADD showed small hyperautofluorescent dots originating in hyperreflective photoreceptor debris at or above the RPE on FAF imaging. FAF further showed large hyperautofluorescent spots corresponding to sub-RPE drusen in both ADD and BD-ADD. The tissue-specific contrast of PS-OCT imaging allowed identification of the RPE within the macular lesion, structural changes of the subretinal material and incipient scar formation. Genetic analysis identified pathogenic mutations in both the BEST1 and the EFEMP1 gene in both BD-ADD cases. **Conclusions**: The characteristic morphological and functional features of both diseases are evident in the patients with the BD-ADD dual genotype. The coexistence of two independent autosomal dominant disorders provides an illustrative opportunity to inform our understanding of BEST1- and EFEMP1-mediated retinal disease.

## 1. Introduction

Macular dystrophies (MDs) are a group of inherited retinal disorders characterized by bilateral central visual loss caused mostly by progressive macular atrophy [1]. Best disease (BD) and autosomal dominant drusen (ADD) are two subtypes of monogenic MDs with a distinct retinal phenotype.

BD is a dominantly inherited disorder associated with a variant in the *BEST1* (OMIM #607854) gene [2]. The gene is expressed in retinal pigment epithelium (RPE) cells and encodes bestrophin-1, a protein which forms oligomeric Ca^2+^-gated chloride channels [3,4,5]. Abnormal bestrophin-1 function gives rise to changes in pH in the RPE which leads to impaired fluid transport of these cells [6]. Physical separation of photoreceptor layers from the RPE is caused by resulting subretinal fluid which leads to progressive accumulation of lipofuscin due to shedding of outer segment discs without proper phagocytosis by the RPE cells [7]. The characteristic appearance of BD is a round, bilateral, dome-shaped macular lesion containing yellowish-white material, which over time can undergo resorption and progression resulting in atrophy, fibrosis or choroidal neovascularization [8]. Mutations of the *BEST1* gene can also give rise to other phenotypes such as autosomal recessive bestrophinopathy (ARB) [9], adult-onset vitelliform macular degeneration [10], and autosomal dominant vitreoretinochoroidopathy [11].

ADD is associated with variants in the *EFEMP1* gene (EGF-containing fibulin-like extracellular matrix protein-1; OMIM #601548) and encompasses the phenotypes “Doyne honeycomb retinal dystrophy” and “Malattia Leventinese” [1,12]. EFEMP1 is a member of the fibulin family of proteins, extracellular matrix proteins that serve a structural role and are involved in cell signalling [13,14]. They play a role in cell growth, adhesion, and motility because of their close contact to cell surface receptors [14]. Improper folding of the EFEMP1 protein may prevent efficient secretion from cells [15] leading to protein accumulation within and beneath the RPE cells overlaying the drusen present in the disorder without being a major component of the drusen themselves [16]. Clinical features include radially orientated elongated yellowish-white drusen in the macula that develop in early adult life and may include areas of the optic disc. In most cases vision loss occurs later in life due to degeneration of RPE and outer retinal layers and eventual formation of choroidal neovascularization [17].

Comprehensive clinical investigations revealed the presence of both BD and ADD in a mother and daughter with confirmed pathogenic variants in the *BEST1* and the *EFEMP1* genes. The study details the functional and morphologic characteristics using advanced imaging technology and compares the retinal findings to a patient with isolated ADD. The co-occurrence of pathogenic variants in two independent genes, each individually sufficient to cause a distinct autosomal dominant macular dystrophy, raises a number of biologically intriguing questions that are inherently difficult to address. Both BEST1 and EFEMP1 act within or adjacent to the RPE–Bruch’s membrane complex, yet through largely distinct mechanisms (ion channel dysfunction and impaired fluid transport in the case of BEST1, versus extracellular matrix protein misfolding and accumulation in the case of EFEMP1) making it unclear a priori whether their combined presence would simply produce a superimposition of two separate phenotypes, or whether shared downstream pathways (e.g., RPE stress, impaired phagocytosis, or Bruch’s membrane remodelling) could result in genuine interaction or mutual modification of disease expression.

## 2. Patients and Methods

Three patients of Austrian Caucasian ancestry were ascertained from the inherited retinal disease unit of the Department of Ophthalmology, Medical University of Vienna: the affected daughter (patient 1) and mother (patient 2), and one unaffected son of patient 1. A sister of patient 1 was apparently similarly affected but not available for examination. No consanguinity was recorded.

In addition, one patient with phenotypically and genotypically confirmed ADD was included for comparison (patient 3).

This study adhered to the tenets of the Declaration of Helsinki and was approved by the local ethics committee of the Medical University of Vienna (EK1594/2018). Informed written consent was obtained from all patients prior to the start of study.

### 2.1. Clinical Investigations and Electrophysiology

The patients underwent a complete ophthalmic examination including best-corrected visual acuity (BCVA), applanation tonometry, slit-lamp biomicroscopy and dilated fundus examination. Full-field and pattern electroretinography (ERG, PERG), and electrooculography (EOG) were performed to incorporate the International Society for Clinical Electrophysiology of Vision Standards [18,19,20].

### 2.2. Conventional Retinal Imaging

Retinal fundus images in patients 1 and 3 were acquired using an ultra-wide-field (133°) true colour fundus camera (Clarus 700, Carl Zeiss Meditec, Jena, Germany). Further fundus photographs and green light fundus autofluorescence (FAF) images were obtained in all patients by ultra-wide-field (200°) confocal laser scanning ophthalmoscopy (Optos). Spectral-domain optical coherence tomography (OCT) and blue-light FAF imaging (50°) was performed in all patients using a HRA + OCT Spectralis (Heidelberg Engineering, Heidelberg, Germany).

### 2.3. Polarization-Sensitive Optical Coherence Tomography Imaging

Polarization-sensitive optical coherence tomography (PS-OCT) imaging was performed in patients 1 and 3 using a high-speed PS-OCT system with retinal tracker developed at the Center for Medical Physics and Biomedical Engineering at the Medical University of Vienna. The principles of PS-OCT and the instrument used in this study have been described previously [21,22,23]. The system is capable of measuring the intensity of the backscattered light (as in standard SD-OCT imaging), the degree of polarization uniformity (DOPU) [24], phase retardation (introduced by tissue birefringence) and birefringent optic axis orientation (corresponding to the orientation of birefringent structures such as fibrotic tissue) [25]. Retinal layers can therefore be differentiated into polarization-preserving (e.g., photoreceptors), depolarizing (e.g., RPE cells) and birefringent layers (e.g., retinal nerve fibre layer, Henle fibre layer, fibrotic tissue) [21,26,27]. The main source of depolarization observed from the RPE layer is melanin-containing components (e.g., melanosomes in RPE cells) [28]. Based on this contrast mechanism, PS-OCT has the unique ability to specifically visualize the RPE layer or remnants thereof, even in cases where the normal retinal structures are heavily distorted [29,30]. Colour-coded B-scans, displaying DOPU, retardation and axis orientation were generated for visualization of the polarization-sensitive parameters. In addition, en-face maps were created at the level of drusen-like deposits and photoreceptor layers. Volume scans consisting of 1024 × 250 A-scans and covering an area of approximately 8.4 × 6.3 mm were acquired in 4.5 s. Motion artefacts are supressed through hardware-based retinal tracking [23].

### 2.4. Molecular Genetic Analysis

Genomic DNA was isolated from peripheral blood lymphocytes. Genetic testing was performed using next-generation sequencing, on a NextSeq500 using the TruSight™ Exome Library Preparation Kit according to the instruction of the vendors (Illumina, San Diego, CA, USA). Overall, above 90% of the sequences were analyzed with at least 10-fold coverage. The analysis of the data was performed with the commercial software VarSeq (Golden Helix^®^, Bozeman, MT, USA, Version 1.5.0). The pathogenicity of variants was evaluated according to ACMG (American College of Medical Genetics and Genomics) guidelines [31]. CNV analysis of the analyzed sample was performed using the VarSeq CNV module (Golden Helix^®^) with in-house reference BAM files. The pathogenic variants detected by next-generation sequencing were confirmed by Sanger sequencing. The carrier status of patient 2 for the familial variants was confirmed by Sanger sequencing.

## 3. Results

### 3.1. Patient 1

**Figure 1:**
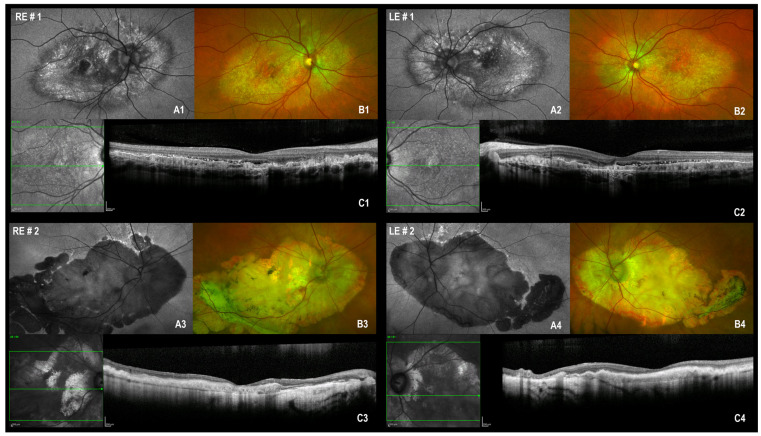
Patient number 1 is shown at the top. Her mother (patient 2) is shown at the bottom. Fundus autofluorescence (**A1**,**A2**) showing a generalized reduction in macular FAF, with spots of increased FAF corresponding to the confluent drusen in a radial placement in the macula and at the optic disc margins as seen in the fundus photograph (**B1**,**B2**). Central OCT B-scan (**C1**,**C2**) depicts large hyperreflective foci corresponding to sub-RPE drusen-like deposits in the thickened RPE–Bruch’s membrane complex and numerous small hyperreflective dots anterior to the RPE. In the subfoveal area, detachment of the neurosensory retina, accumulation of subretinal debris and “stalactitic” elongation of photoreceptor outer segments is visible. FAF (**A3**,**A4**) showing large areas of hypoautofluorescence with some hyperautofluorescence at the border of the lesion in both eyes. Fundus photography (**B3**,**B4**) shows large yellowish macular lesions extending the vascular arcades with marked RPE atrophy and pigment migration. Central OCT B-scan (**C3**,**C4**) depicts massive deposition of hyperreflective material between Bruch’s membrane and the substantially thinned retina. The green line indicates the position of B-scans.

This 50-year-old woman had light sensitivity and progressive loss of visual acuity noted at the age of 45. BCVA was 20/63 in each eye. The patient was mildly hyperopic. Slit-lamp examination showed a normal anterior segment with a clear visual axis. The intraocular pressure was 14 mmHg in the right and 15 mmHg in the left eye. Fundus examination revealed tightly packed yellow-whitish drusen in the macula in a radial pattern including the peripapillary region, and RPE changes in both eyes (Figure 1). The peripheral retinae were unremarkable.

#### 3.1.1. Electrophysiology

All full-field ERGs fell well within the normal range. PERGs showed severe P50 reduction in both eyes, indicating severe macular dysfunction. There was marginally subnormal EOG light rise (Arden index 160% in the right eye, 170% in the left eye) [see Supplementary Materials, S3-FF_1, S5-PERG_1, S1-EOG_1].

#### 3.1.2. Retinal Imaging

Macular SD-OCT B-scans showed separation of Bruch’s membrane and RPE by extensive dense, highly reflective material in both eyes. Detachment of the neurosensory retina, accumulation of subretinal debris and “stalactitic” elongation of photoreceptor outer segments was bilaterally present in the parafoveal area. Inner retinal layers and the foveal depression were well preserved. The large yellow-whitish drusen corresponded to localized elevations of the RPE–Bruch’s membrane complex (Figure 1).

**Figure 2:**
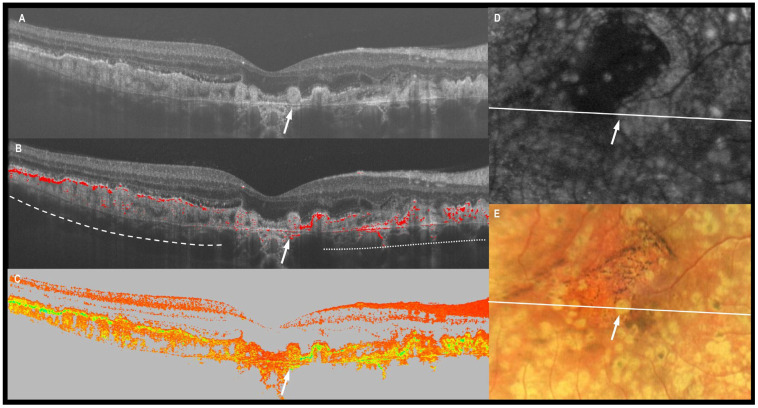
PS OCT images of patient 1: Intensity-based B-scan (**A**), RPE segmentation B-scan (**B**) and DOPU image (**C**). The white line indicates the position of B-scans on FAF (**D**) and CF images (**E**). The RPE segmentation B-scan allows for identification of the RPE within the deposition of hyperreflective material between Bruch’s membrane and the outer retina. In the temporal macula, the RPE appears to be located on top of the complex (dashed line) whereas in the nasal macula, RPE cells seem to be disseminated within (dotted line). The white arrows indicate a characteristic hyperreflective (**A**), hyperautofluorescent (**D**) yellowish (**E**) ADD-like druse containing RPE remnants.

**Figure 3:**
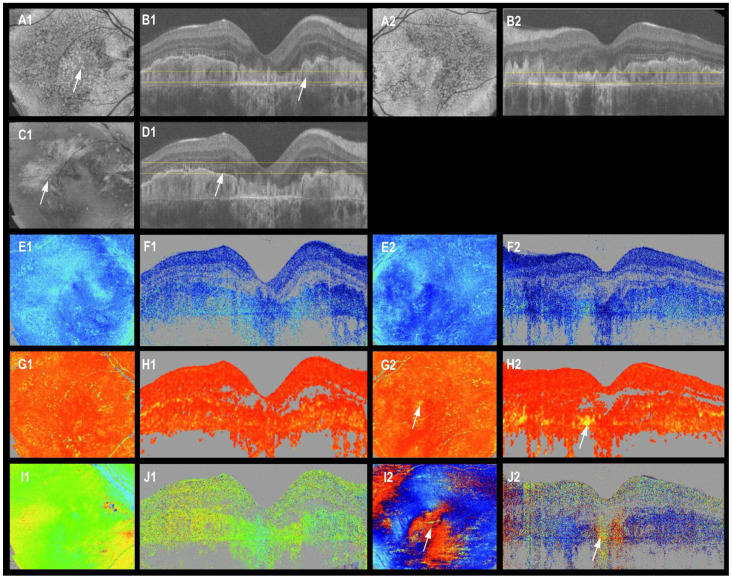
Patient 1. PS OCT images: Intensity-based images (**A1**–**D2**). The top row shows the confluent mass of subretinal material with a relatively smoother inner surface interface (**B1**,**B2**) and en-face slab images (depth integrated over the region between the horizontal yellow lines indicated in the B-scans) revealing distinct column like organization (Arrow in (**A1**,**A2**,**B1**,**B2**)). In the second row, elongated photoreceptor outer segments (arrows in (**C1**,**D1**)) can be seen in the B-scans and en-face slab of the anterior layers (**C1**). Retardation-based images (**E1**,**E2**,**F1**,**F2**) and birefringence maps (**I1**,**I2**,**J1**,**J2**) identify the presence of large fibrosis (scar) tissue patterns (white arrow in (**I2**,**J2**)) in both eyes (indicated by increased retardation and homogenous axis orientation from areas below the photoreceptor layers). Note the different axis orientation of the scar tissue compared to the nerve fibres (that are birefringent as well). The DOPU images (**G1**,**G2**,**H1**,**H2**) delineate remnants of the RPE or pigments (yellow spots, some are indicated by arrows) that are mainly distributed anterior and posterior to the scar lesions. A DOPU value of 1 indicates polarization-preserving or birefringent tissue, lower DOPU values indicate depolarization. Pixels with intensity values below a certain threshold are displayed in grey. The horizontal lines in the B-scans indicate the depth extension of the adjacent en-face slabs, while the horizontal lines in the en-face images indicate the position of the B-scan. The numbers 1 and 2 indicate the right and left eye, respectively.

FAF imaging showed a heavily distorted macular autofluorescence (AF), with locations of absent AF and increased AF. The latter corresponded to the confluent drusen in a radial placement. However, not all drusen seen on fundus photography were associated with increased AF. Some, e.g., in the presence of RPE atrophy at the centre of the macula, displayed absent or reduced AF. This is consistent with PS-OCT findings within DOPU images showing a variable amount of RPE remnants within the drusen and surrounding atrophic regions (some remnants are marked with arrows in Figure 2 and Figure 3(G2,H2)). Small foci of increased AF were detected between the drusen that corresponded to accumulation of material at or above the RPE on SD-OCT and PS-OCT imaging. There were also foci of increased AF at the optic disc margins, representing peripapillary drusen. The parafoveal fibrotic lesion and areas with RPE hyperplasia appeared hypoautofluorescent. At the photoreceptor level, elongated remnants of cones or rod outer segments are present in the SD-OCT and PS-OCT B-scans respectively. A corresponding en-face slab visualizes these very tiny and highly scattered dots that are only visible temporal to the fovea (cf. Figure 3(C1,D1)). In the accumulated material posterior to the remnants of the RPE, a well-defined axis orientation (and a slight increase in retardation) is present in both eyes, a strong indicator of fibrosis [27]. An additional fibrosis location (with differing axis orientation) is visible only in the left eye (indicated by an arrow in Figure 3(J1,J2)).

**Figure 4:**
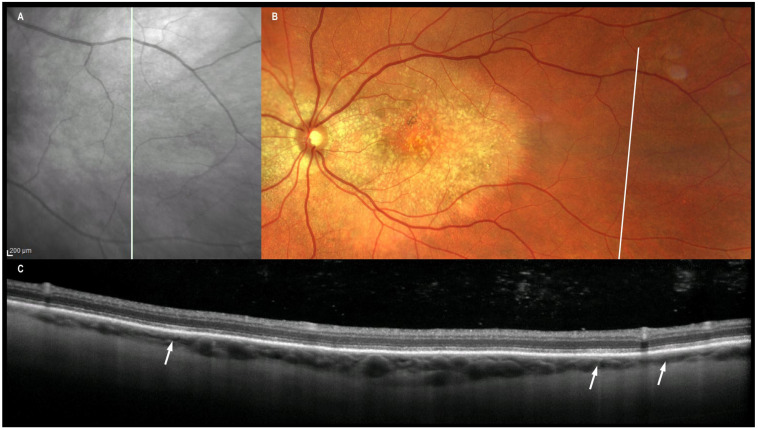
Patient 1. (**A**) Retinal SD-OCT en-face IR image; the white line indicates the position of the B-scans of SD-OCT (**C**) in IR (**A**) and CF images (**B**). The white arrows point to segmental outer layer opacification in the retinal periphery. (**B**) shows the corresponding fundus image.

Mid-peripheral SD-OCT scans showed a thickening of Bruch’s membrane under spots—SOLO (segmental outer layer opacification) (Figure 4).

### 3.2. Patient 2

The 79-year-old mother of patient 1 reported progressive vision loss predominantly in the left eye, starting at the age of 50. BCVA was 20/200 in the right and hand motions in the left eye. Slit-lamp examination showed a mild posterior subcapsular cataract in both eyes. The intraocular pressure was 19 mmHg in the right eye and 20 mmHg in the left eye. Fundus examination showed widespread macular RPE atrophy and fibrosis surrounded by few white retinal deposits and areolar atrophy in the mid-periphery. The optic disc showed a cup/disc ratio of 0.9 and a markedly reduced rim thickness in both eyes consistent with advanced glaucoma.

#### 3.2.1. Electrophysiology

The dim flash dark-adapted ERGs (DA0.01) showed a bilaterally subnormal b-wave with the bright flash dark-adapted ERGs (DA10.0) having markedly subnormal a-waves confirming the defect to be at the level of the rod photoreceptors. Photopic flicker and single flash ERGs were bilaterally delayed and subnormal, consistent with generalized retinal cone system dysfunction. PERGs showed severe reduction in both eyes, indicating severe macular dysfunction. Due to limited fixation capabilities and compliance, EOG could not be performed.

#### 3.2.2. Retinal Imaging

Macular SD-OCT B-scans showed massive deposition of highly reflective material separating Bruch’s membrane and the substantially thinned inner retina, without visible outer retinal layers. There were no visible ADD-like drusen (Figure 1).

PS-OCT could not be performed in this patient.

### 3.3. Patient 3

This 68-year-old patient reported progressive vision loss beginning at the age of 50 years. BCVA was 20/160 in the right eye and 20/32 in the left. Ocular pressure was 11 mmHg in both eyes. Slit-lamp examination showed mild cataracts in both eyes. Fundus examination showed tightly packed yellow-whitish drusen in the central macula in a radial pattern and in the peripapillary region in both eyes. The peripheral retinae appeared normal.

#### 3.3.1. Electrophysiology

Full-field ERGs fell well within the normal range. PERGs showed severe P50 reduction in both eyes, indicating severe macular dysfunction. A normal EOG light rise was present in each eye (Arden index 320% right, 360% left) [see Supplementary Materials, S4-FF_2, S6-PERG_2, S2-EOG_2].

#### 3.3.2. Retinal Imaging

**Figure 5:**
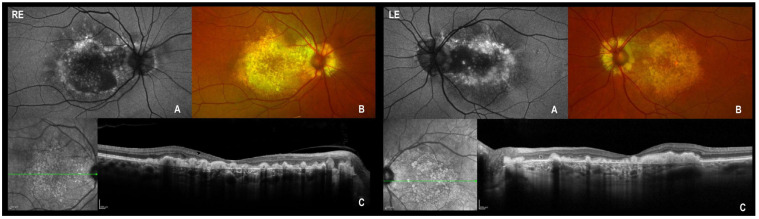
Patient 3 (EFEMP1) fundus autofluorescence (**A**) showing a generalized reduction in macular FAF, with spots of increased FAF corresponding to the confluent drusen in a radial placement in the macula and at the optic disc margins as seen in the fundus photograph (**B**). Central OCT B-scan (**C**) shows large hyperreflective spots in a saw-toothed pattern corresponding to sub-RPE drusen-like deposits in the thickened RPE–Bruch’s membrane. The green line indicates the position of B-scans.

Macular SD-OCT B-scans showed a separation of Bruch’s membrane and RPE by extensive dense, highly reflective material in both eyes. Inner retinal layers were preserved, as was the foveal depression. The large yellow-whitish drusen corresponded to localized elevations of the RPE–Bruch’s membrane complex in a saw-toothed pattern (Figure 5).

**Figure 6:**
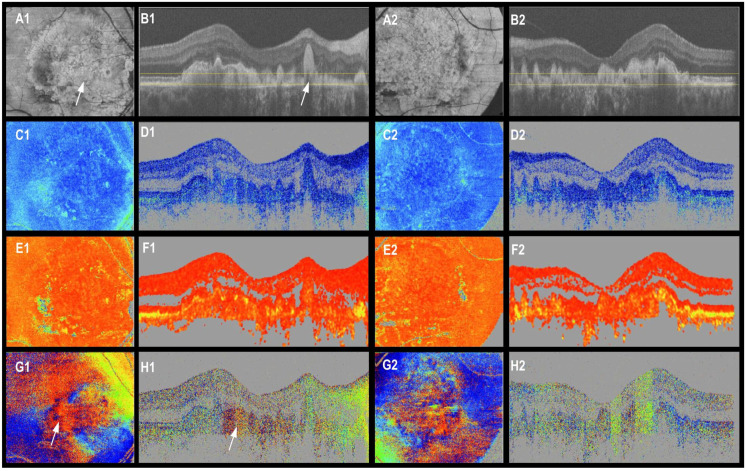
Patient 3 (EFEMP1). The top row shows the typical saw-toothed pattern of subretinal material (arrows in (**A1**,**B1**)) in B-scans (**B1**,**B2**) and en-face slab images (**A1**,**A2**). Retardation images (**C1**,**C2**,**D1**,**D2**) and thresholded DOPU images (**E1**,**E2**,**F1**,**F2**) delineating the location and border of the RPE where, similar to patient 1, temporal RPE seems largely preserved and nasal RPE is distributed throughout the subretinal debris. Birefringence maps (**G1**,**G2**,**H1**,**H2**) identify scar tissue patterns (arrows in (**G1**,**H1**)) in both eyes. Yellow lines depict the depth of en-face slab images (**A1**,**A2**).

FAF imaging showed generalized low levels of macular AF, with spots of increased AF corresponding to the confluent drusen in a radial placement. As in patient 1, not all the drusen identified on fundus photography were associated with increased AF. This is consistent with PS-OCT findings showing a variable amount of RPE remnants within the drusen (Figure 6). In addition, depolarizing structures are visible at the level of Bruch’s membrane, suggesting that remnants of the RPE are remaining at this location, in contrast to the observation in patient 1. The en-face slabs show dome-shaped structures similar to those in patient 1. However, the elongated photoreceptors are not evident. There were also foci of increased AF at the optic disc margins, which represent peripapillary drusen, though markedly less so than in patient 1. The presence of fibrosis is also less pronounced than in patient 1 and mainly visible in the right eye (Figure 6(G1,G2,H1,H2)).

#### 3.3.3. Genetics

Analysis of the whole-exome sequencing data revealed the presence of two heterozygous variants in the *EFEMP1*-gene (NM_001039348.2:c.1033C > T, NP_001034437.1: p.Arg345Trp) and in the *BEST1*-gene (NM_004183.3: c.85T > C, NP_004174.1: p.Tyr29His) in patients 1 and 2, which have previously been reported [32,33,34,35]. The presence of both variants was confirmed by Sanger sequencing. Analysis of the ADD patient revealed the presence of the same *EFEMP1*-gene variant (NM_001039348.2:c.1033C > T, NP_001034437.1: p.Arg345Trp) as the patients with dual pathology.

## 4. Discussion

This report describes the clinical and genetic features of a mother and daughter harbouring pathogenic variants in both the *BEST1* and *EFEMP1* genes, usually associated with Best disease and autosomal dominant drusen respectively. No report of similar cases has been identified. The findings are compared with those in a single variant *EFEMP1* patient.

The initial clinical diagnosis in both patients was ADD, since the appearance of the maculopathy was dominated by the radially oriented yellow-whitish macular and parapapillary drusen. However, once the molecular defects had been ascertained, characteristic features of both ADD and BD were identified upon re-examination of the phenotype. In addition to conventional functional and morphological assessment, advanced imaging technology including PS-OCT (polarization-sensitive optical coherence tomography) provided in-depth analysis of retinal changes in these unique cases of *BEST1*- and *EFEMP1*-mediated disease.

Consistent with previous OCT studies in ADD [36,37,38], SD-OCT images in both patients showed a saw-toothed pattern in the thickened RPE–Bruch’s membrane complex corresponding to the characteristic macular drusen-like deposits. Histopathologic studies of postmortem donor eyes and knock-in mice carrying the heterozygous and homozygous p.R345W mutations provided insight into structural changes in ADD [16,39]. The misfolded EFEMP1 protein is secreted inefficiently and accumulates within and under the RPE, and it is the aberrant accumulation of EFEMP1 that underlies the formation of the extensive, highly reflective material in the RPE–Bruch’s membrane complex.

PS-OCT provides a tissue-specific contrast and thus allowed definite identification of RPE remnants in respect to the highly reflective material. PS-OCT displays the variable localization and condition of the RPE which is not well reflected in CF images. Axis orientation maps were derived since the analysis of fundus photographs precludes the unambiguous detection of scar formation within macular lesions in both BD-ADD and ADD. These maps showed that birefringence is introduced posterior to the photoreceptor band, indicating the presence of fibrosis. It should however be noted that both RNFL and Henle fibre also introduce birefringence. Specifically, the location of the RNFL is visible in the axis orientation images (Figure 3(I1,I2,J1,J2) and Figure 6(G1,G2,H1,H2)) as these show a different orientation compared to the fibrotic tissue. It is well known that Henle fibre extends radially from the fovea resulting in a typical axis orientation pattern in the macula [26] not evident in the two cases described. This underlines the suggestion that the homogeneous axis orientation indeed indicates the presence of fibrosis. The vertical stripe artefacts in the axis orientation images arise from problems in the retinal surface detection necessary for compensating the data for anterior segment birefringence [40].

FAF imaging in both patient 1 and 3 demonstrated large hyperautofluorescent spots corresponding to sub-RPE drusen as previously described in ADD [17,41]. The increased AF associated with the drusen in ADD contrasts with that in the drusen of age-related macular degeneration (AMD), where there is usually little correspondence between the distribution of drusen and FAF, although large soft foveal drusen may be associated with increased FAF [42]. The hyperautofluorescence associated with drusen is therefore an important feature in the differential diagnosis of ADD [17]. The radial appearance of ADD, which is also visible in the reported cases, is established.

In contrast, numerous small hyperautofluorescent foci do not occur in ADD but may be a feature of BD. Correlation with SD-OCT indicates they originate in hyperreflective dots at or above the RPE, similar to the photoreceptor abnormalities seen in ARB that are reflected by changes including “stalactite”-like thickening and elongation [43]. These findings might indicate dysfunction, i.e. limited phagocytotic activity leading to accumulation of photoreceptor debris on top of the RPE. These features are unlikely to represent nonspecific late-stage photoreceptor degeneration or scarring alone, as elongated photoreceptor outer segments, subretinal hyperreflective debris, and small hyperautofluorescent/hyperreflective foci have been specifically and repeatedly described in BD and ARB in prior multimodal imaging series [43,44], whereas they are not a recognized feature of isolated EFEMP1-associated ADD, in which drusen formation instead reflects extracellular accumulation of misfolded protein within and beneath an intact RPE [16]. Indeed, none of these three features were observed in our isolated EFEMP1 comparator (patient 3), further supporting their specificity to the BEST1-associated component of the phenotype rather than representing nonspecific degenerative change. The subretinal location of presumed lipofuscin-containing debris and vitelliform material in BD, which appears hyperautofluorescent on FAF imaging, has previously been reported [43].

Witsberger et al. consistently observed diffuse whitening surrounding the arcade vessels and in the (mid-) periphery of patients with ARB on fundoscopy and in fundus photography and FAF [44]. Such changes correspond to a distinct hyperreflectivity of the EZ and the photoreceptor outer segment layer on OCT, which was not linked to fluid accumulation. They suggested that this diffuse outer layer opacification (DOLO) signifies an involvement of the entire RPE by ARB-linked mutations of the *BEST1* gene. The present patients, although not visible on fundoscopy or in fundus photography, showed similar EZ and photoreceptor outer segment layer opacification on OCT in the mid-periphery. Moreover, it was visible only in small proportions of cross-sectional OCT scans, and the term segmental outer layer opacification (SOLO) seems a more appropriate descriptive term. To date, neither DOLO nor SOLO has been described in BD, in which, unlike the progressive photoreceptor degeneration of ARB, generalized retinal photoreceptor dysfunction does not occur [45]. It is therefore tempting to speculate that the two distinct molecular pathologies present in the two patients might interact. A diffusion barrier may be created by the *EFEMP1*-associated thickening of Bruch’s membrane leading to RPE dysfunction and subsequently loss of the normal RPE/photoreceptor layering as in ARB.

Despite the marked intrafamilial and interfamilial variability, both BD and ADD are associated in most patients with a slow central visual deterioration and a relatively good prognosis unless complicated by a secondary CNV [17,46]. Neither is associated with generalized retinal photoreceptor dysfunction and abnormal ERGs. Both patients were asymptomatic until the fifth decade of life, when they developed reduced visual acuity. ADD patients typically develop symptoms at the same age or earlier in the fourth decade [17], which suggests that the dual pathology does not appear to markedly enhance progression of the disease. Electrophysiological testing showed macular dysfunction in both patients, in keeping with either BD or ADD [47]. Dark- and light-adapted ffERG responses were normal in patient 1 but indicated generalized rod and cone dysfunction in patient 2. Detailed perimetry performed on ADD patients suggests widespread retinal dysfunction not confined to the macula [17]. However, previously reported electrophysiological findings in ADD showed normal scotopic responses but borderline delayed or reduced 30 Hz flicker responses [38,47]. Patient 1 had a very mildly subnormal EOG that could well simply reflect the difficulties of recording EOG in a patient with central visual loss, but is certainly not suggestive of generalized RPE dysfunction. Patient 2 did not have EOG. In BD the EOG is often flat and nearly always <120%. There is no evidence in our two patients of generalized RPE dysfunction, but a normal EOG can very rarely be found in a patient with BEST1 variant [48]. It should be emphasized that the generalized rod and cone dysfunction observed on full-field ERG in patient 2 cannot be unambiguously attributed to the retinal dystrophy alone, given her concurrent advanced glaucoma, which may independently affect ERG and visual function; the relative contribution of each condition to her electrophysiological profile cannot be disentangled in this study. More broadly, the electrophysiological data in both patients provide only limited support for BEST1-mediated dysfunction, in contrast to the more consistent support derived from multimodal imaging and genotype. We therefore consider the diagnosis of dual BD-ADD pathology in this family to rest primarily on imaging and genetic findings, with electrophysiology contributing corroborative rather than primary evidence.

Both patients harboured disease-causing variants in both the *BEST1* and the *EFEMP1* gene. A single heterozygous missense variant, p.Arg345Trp, in *EFEMP1* is reported to cause ADD [32,33,34]. The same variant was present in our 2 patients, which supports the pathogenic role of *EFEMP1* in these cases. Predictions of genotype–phenotype relations are more complex in *BEST1* disease, since there are various possible phenotypes [49]. In most BD cases, missense variants in the first half of the *BEST1* gene are identified. The current data show the previously described heterozygous missense mutation (c.85T > C) resulting in the amino acid change (p.Tyr29His) located within a defined hotspot region of the *BEST1* gene [34]. The classification of the BEST1 c.85T > C (p.Tyr29His) variant as pathogenic in this family is supported by its prior identification in clinically diagnosed Best disease patients and its location within a recognized mutational hotspot region of the gene [34], together with the BD-like imaging features observed here, rather than by functional electrophysiological confirmation, since EOG support was weak in patient 1 and unobtainable in patient 2. We therefore consider the pathogenic role of this variant to be well supported by genotype, prior clinical reports, and imaging evidence, while acknowledging that segregation data in additional affected relatives and functional channel studies are lacking in this family.

This study has several limitations. First, the sample size is very small, comprising only two related patients with the dual BD-ADD genotype and a single comparator patient with isolated ADD; consequently, the findings reported here are descriptive and hypothesis-generating rather than confirmatory, and conclusions regarding genotype–phenotype correlation cannot be generalized. Second, segregation analysis was incomplete: the unaffected son of patient 1 was not genetically tested, and the similarly affected sister of patient 1 was not available for examination, precluding a full assessment of co-segregation of the two variants with the phenotype. Third, no longitudinal follow-up data are available, so the natural history and rate of progression of the dual pathology compared to either disease in isolation cannot be assessed. Fourth, electrophysiological support for BEST1-mediated dysfunction was limited, as patient 1 showed only a mildly subnormal EOG and patient 2 was unable to complete EOG testing; the diagnosis of BD in these patients therefore relies more heavily on imaging and genotype than on functional electrophysiological confirmation. Fifth, advanced glaucoma and mild cataract in patient 2 represent potential confounders for the interpretation of her visual function and electrophysiological findings. Finally, only a single comparator patient with isolated ADD was available; while this patient was deliberately chosen because she carries the identical EFEMP1 variant found in patients 1 and 2, allowing a genotype-matched comparison, a single comparator cannot capture the known phenotypic variability of isolated ADD, and the findings presented here should therefore be regarded as illustrative rather than representative of isolated EFEMP1-associated disease as a whole.

In conclusion, this is the first descriptive report of patients phenotypically and genotypically diagnosed with both BD and ADD to the best of our knowledge. While the limited sample precludes definitive conclusions, we hypothesize that these cases provide an example of how modifying factors may plausibly act in autosomal dominant MDs, where variability of disease expression frequently occurs and is generally ascribed to the modifying effects of other genetic attributes, environmental factors, or both [1,17]. The future identification of such modifying factors may improve our understanding of the pathophysiology of monogenic MDs and be key to identifying new treatments and interpreting the therapeutic benefits. The genetic analysis using the present strategy of multigene investigation may be helpful in identifying some of the modifiers or compound factors modulating the BEST1 phenotype.

## Data Availability

The original contributions presented in this study are included in the article/Supplementary Materials. Further inquiries can be directed to the corresponding author.

## Supplementary Materials

The following supporting information can be downloaded at: https://www.mdpi.com/article/10.3390/jcm15145495/s1; Electrophysiological Data (Fullfield, PERG and EOGs of Patients 1 and 3).
