# Supplementary material for: Multimodal Imaging of Dual BEST1/EFEMP1-Associated Hereditary Macular Disease
Source: J Clin Med. 2026 Jul 13;15(14):5495. doi: 10.3390/jcm15145495 (PMC13412455; doi:10.3390/jcm15145495)
Supplement: Supplementary file 1 [file jcm-15-05495-s001.zip › EOG_1.pdf]

Saccades at markers:

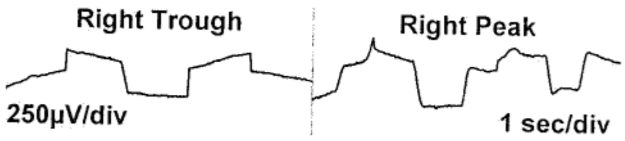

Arden Ratio	Trough	Peak
1,6	232,4µV	380,9µV

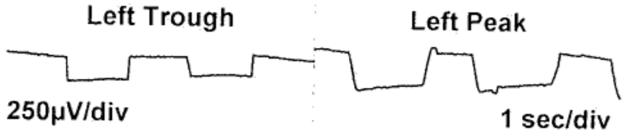

Arden Ratio	Trough	Peak
1,7	186,0µV	310,5µV
