# Supplementary material for: Multimodal Imaging of Dual BEST1/EFEMP1-Associated Hereditary Macular Disease
Source: J Clin Med. 2026 Jul 13;15(14):5495. doi: 10.3390/jcm15145495 (PMC13412455; doi:10.3390/jcm15145495)
Supplement: Supplementary file 1 [file jcm-15-05495-s001.zip › EOG_2.pdf]

Saccades at markers:

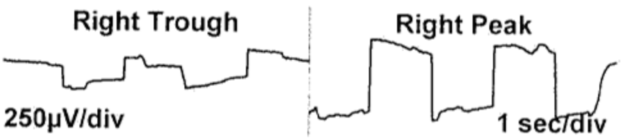

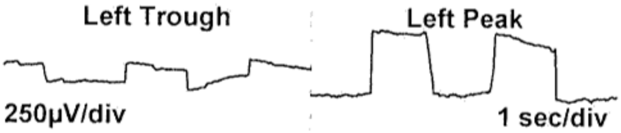

Arden Ratio	Trough	Peak
3,2	6,6μV/deg	21,4μV/deg

Arden Ratio	Trough	Peak
3,6	5,6μV/deg	20,0μV/deg
