# Supplementary material for: Multimodal Imaging of Dual BEST1/EFEMP1-Associated Hereditary Macular Disease
Source: J Clin Med. 2026 Jul 13;15(14):5495. doi: 10.3390/jcm15145495 (PMC13412455; doi:10.3390/jcm15145495)
Supplement: Supplementary file 1 [file jcm-15-05495-s001.zip › FF_1.pdf]

Diagnosis:

Scotopic 0.01 ERG GF

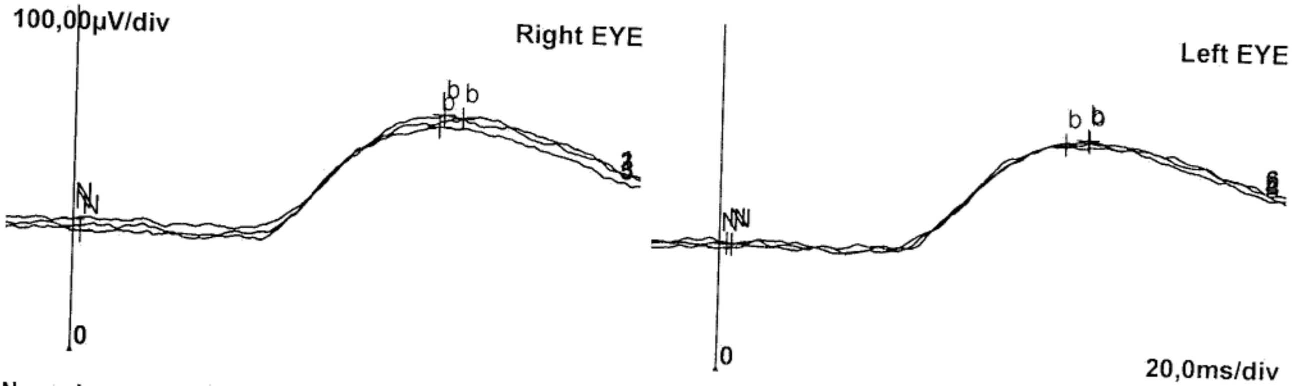

Normals	67-91	95,0µV-305µV
Channel	b [ms]	b-wave
1 R-1	87,2	293µV
3 R-1	91,6 (!)	274µV
5 R-1	86,0	237µV
2 L-2	86,9	254µV
4 L-2	81,3	250µV
6 L-2	86,6	272µV

Scotopic 3.0 ERG GF

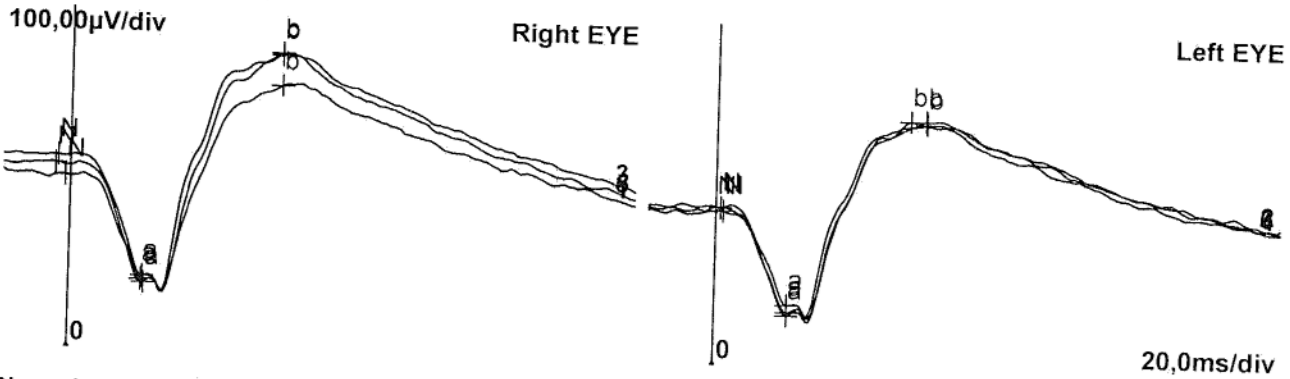

Normals	14-22	33-46	155µV-356µV	290µV-654µV	1,50-2,60
Channel	a [ms]	b [ms]	a-wave	b-wave	b/a
1 R-1	17,3	49,9 (!)	237µV	459µV	1,94
3 R-1	17,9	49,9 (!)	272µV	544µV	2,00
5 R-1	17,6	49,9 (!)	275µV	524µV	1,91
2 L-2	17,3	49,6 (!)	240µV	450µV	1,88
4 L-2	17,3	49,3 (!)	231µV	451µV	1,95
6 L-2	17,3	45,8	223µV	433µV	1,94

Scotopic 10.0 ERG GF

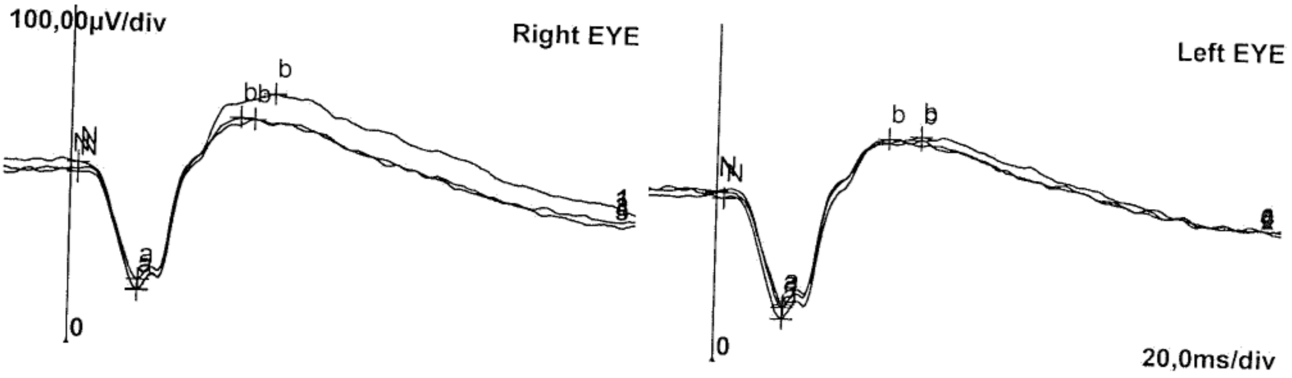

Channel	a [ms]	b [ms]	a-wave	b-wave
1 R-1	16,4	48,1	299µV	472µV
3 R-1	16,4	43,4	273µV	409µV
5 R-1	16,7	40,2	258µV	390µV
2 L-2	16,1	48,1	283µV	441µV
4 L-2	16,1	40,5	271µV	404µV
6 L-2	16,4	48,1	261µV	397µV

Scotopic 3.0 Oscillatory Potential ERG GF

50,00µV/div

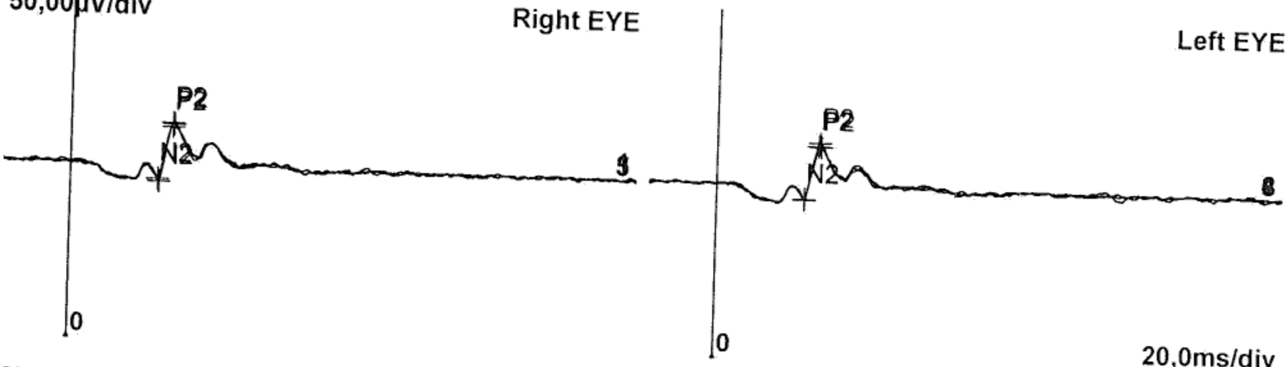

Channel	N2 [ms]	P2 [ms]	OS2
1 R-1	20,8	24,4 (!)	60,2µV
3 R-1	21,1	24,4 (!)	67,9µV
5 R-1	20,8	24,7 (!)	63,2µV
2 L-2	20,8	24,4 (!)	65,4µV
4 L-2	20,8	24,7 (!)	60,2µV
6 L-2	20,8	24,1 (!)	61,7µV

Photopic 3.0 ERG GF

50,00µV/div

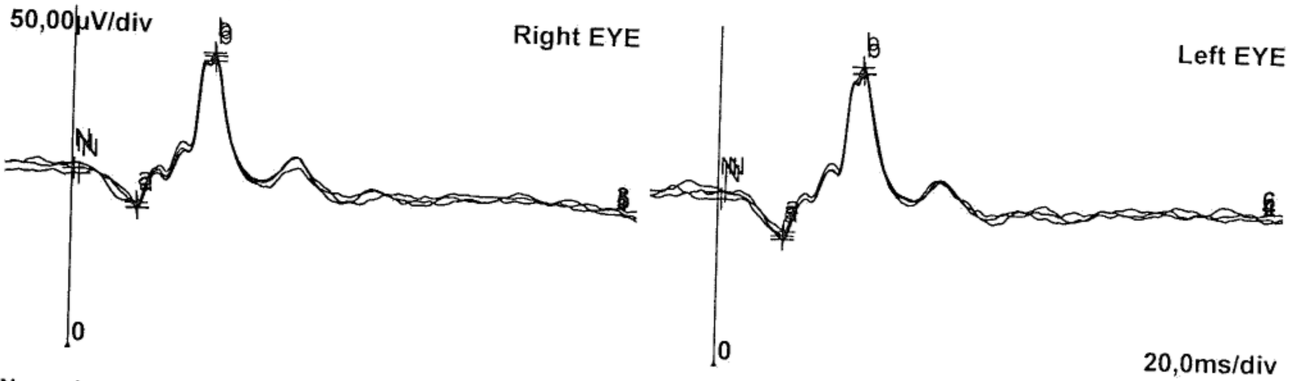

Normals	13-16	29-33	26,0µV-62,0µV	103µV-250µV
Channel	a [ms]	b [ms]	a-wave	b-wave
1 R-1	15,9	33,5 (!)	43,8µV	185µV
3 R-1	15,6	33,8 (!)	44,8µV	176µV
5 R-1	15,9	33,8 (!)	39,3µV	177µV
2 L-2	15,6	33,8 (!)	49,8µV	203µV
4 L-2	15,9	34,1 (!)	45,6µV	191µV
6 L-2	15,6	34,1 (!)	45,9µV	199µV

Photopic 3.0 Flicker 30Hz ERG GF

50,00µV/div

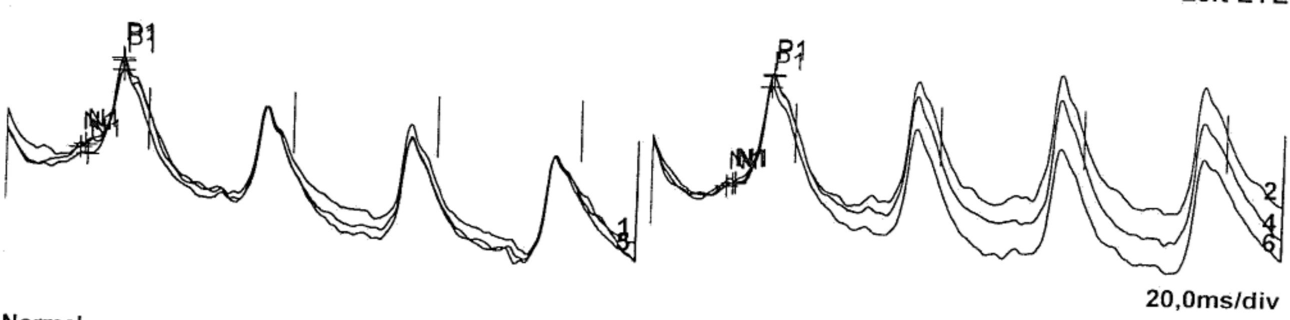

Normals - -

Channel	N1 [ms]	P1 [ms]	N1-P1
**1 R-1**	19,1	27,9	99,9µV
**3 R-1**	17,9	27,6	106µV
**5 R-1**	19,7	27,9	100µV
**2 L-2**	20,0	28,8	127µV
**4 L-2**	17,9	28,8	133µV
**6 L-2**	19,4	28,2	115µV
