# Supplementary material for: Multimodal Imaging of Dual BEST1/EFEMP1-Associated Hereditary Macular Disease
Source: J Clin Med. 2026 Jul 13;15(14):5495. doi: 10.3390/jcm15145495 (PMC13412455; doi:10.3390/jcm15145495)
Supplement: Supplementary file 1 [file jcm-15-05495-s001.zip › FF_2 .pdf]

Diagnosis:

Scotopic 0.01 ERG GF

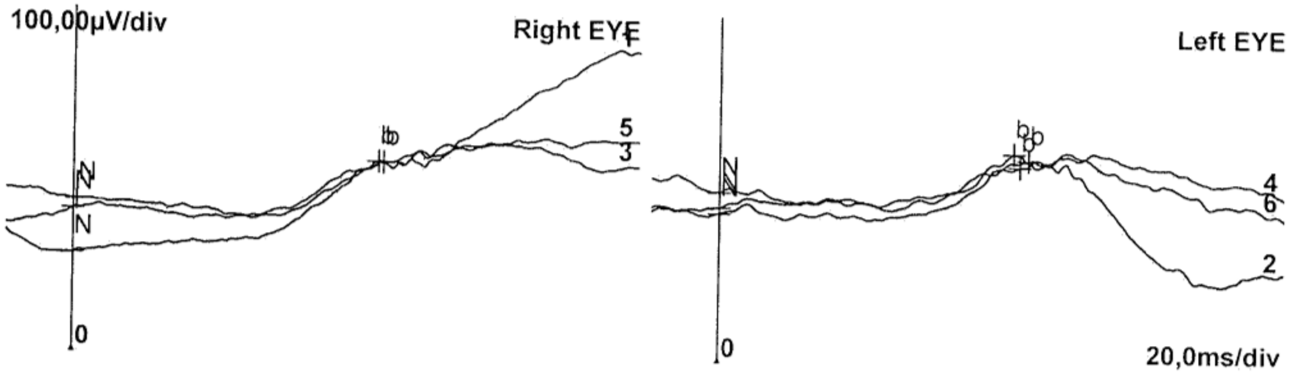

Normals	67-91	95,0µV-305µV
**Channel**	**b [ms]**	**b-wave**
1 R-1	72,2	220µV
3 R-1	73,4	95,5µV
5 R-1	73,4	117µV
2 L-2	69,6	98,9µV
4 L-2	71,0	116µV
6 L-2	73,1	118µV

Scotopic 3.0 ERG GF

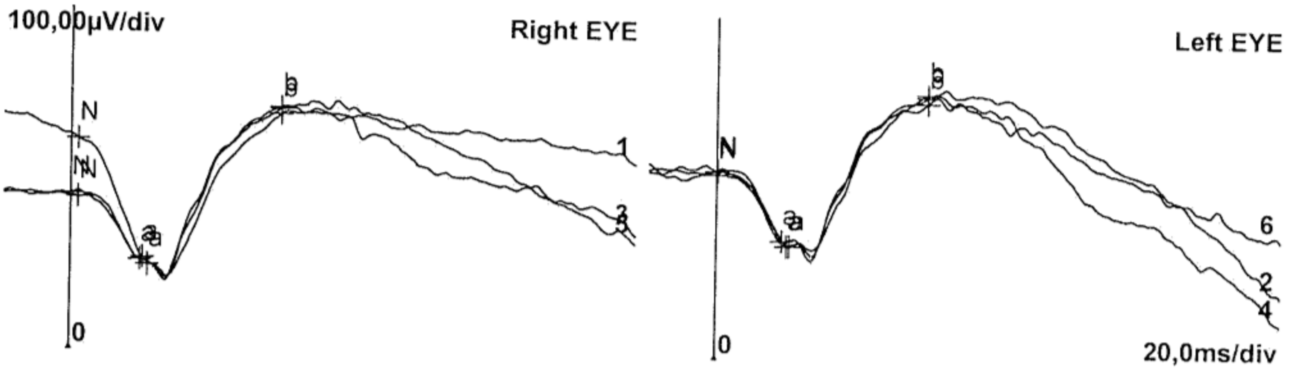

Normals	14-22	33-46	155µV-356µV	290µV-654µV	1,50-2,60
**Channel**	**a [ms]**	**b [ms]**	**a-wave**	**b-wave**	**b/a**
1 R-1	18,5	49,9 (!)	155µV	355µV	2,29
3 R-1	16,7	49,9 (!)	152µV (!)	360µV	2,37
5 R-1	17,3	49,9 (!)	274µV	351µV	1,28 (!)
2 L-2	17,3	49,9 (!)	172µV	357µV	2,08
4 L-2	16,7	49,9 (!)	164µV	351µV	2,14
6 L-2	15,6	49,9 (!)	159µV	323µV	2,03

Scotopic 10.0 ERG GF

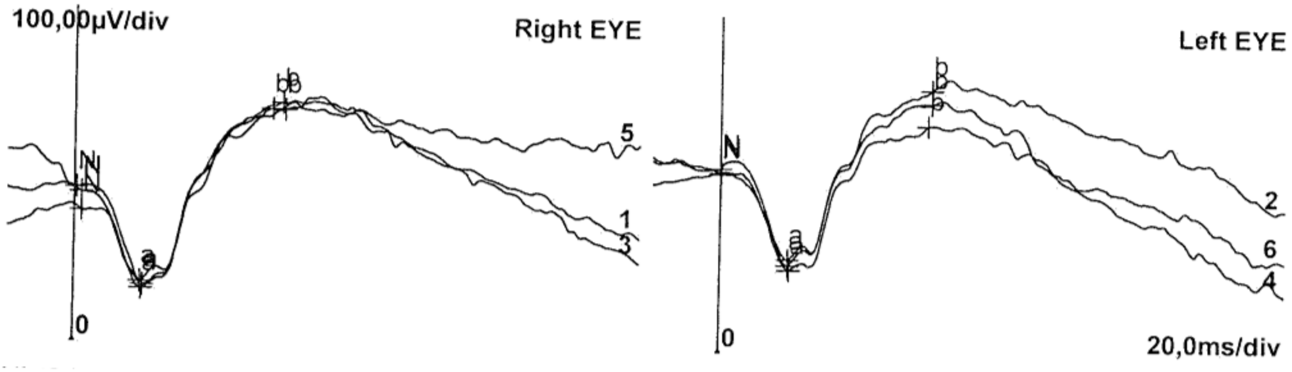

Channel	a [ms]	b [ms]	a-wave	b-wave
**1 R-1**	15,9	49,9	222μV	412μV
**3 R-1**	16,1	49,3	227μV	445μV
**5 R-1**	16,4	47,0	175μV	422μV
**2 L-2**	16,1	49,9	200μV	405μV
**4 L-2**	16,1	49,9	229μV	387μV
**6 L-2**	16,4	49,0	229μV	346μV

Scotopic 3.0 Oscillatory Potential ERG GF

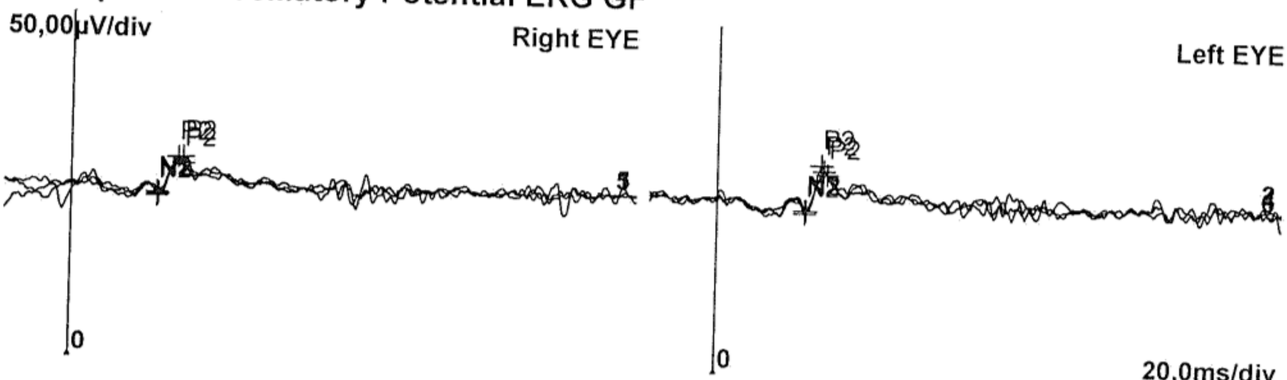

Channel	N2 [ms]	P2 [ms]	OS2
1 R-1	20,5	26,7 (!)	38,6µV
3 R-1	20,8	26,7 (!)	43,9µV
5 R-1	20,5	25,5 (!)	41,5µV
2 L-2	20,8	26,1 (!)	42,0µV
4 L-2	20,8	25,2 (!)	49,3µV
6 L-2	21,1	24,7 (!)	52,7µV

Photopic 3.0 ERG GF

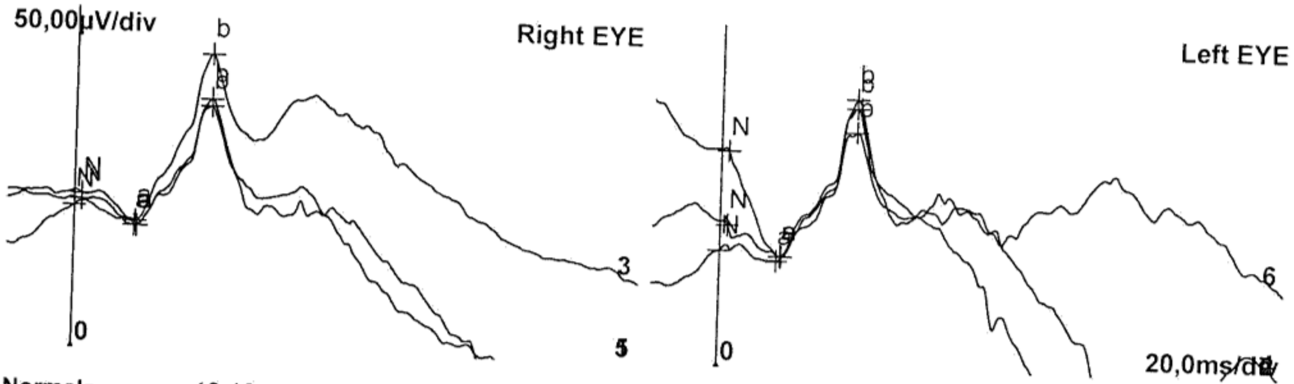

Normals	13-16	29-33	26,0µV-62,0µV	103µV-250µV
Channel	a [ms]	b [ms]	a-wave	b-wave
1 R-1	15,0	32,3	22,3µV (!)	151µV
3 R-1	14,7	32,3	30,7µV	198µV
5 R-1	14,4	32,3	29,3µV	143µV
2 L-2	14,4	32,0	123µV	148µV
4 L-2	13,5	32,0	11,6µV (!)	182µV
6 L-2	14,7	32,0	36,3µV	188µV

Photopic 3.0 Flicker 30Hz ERG GF

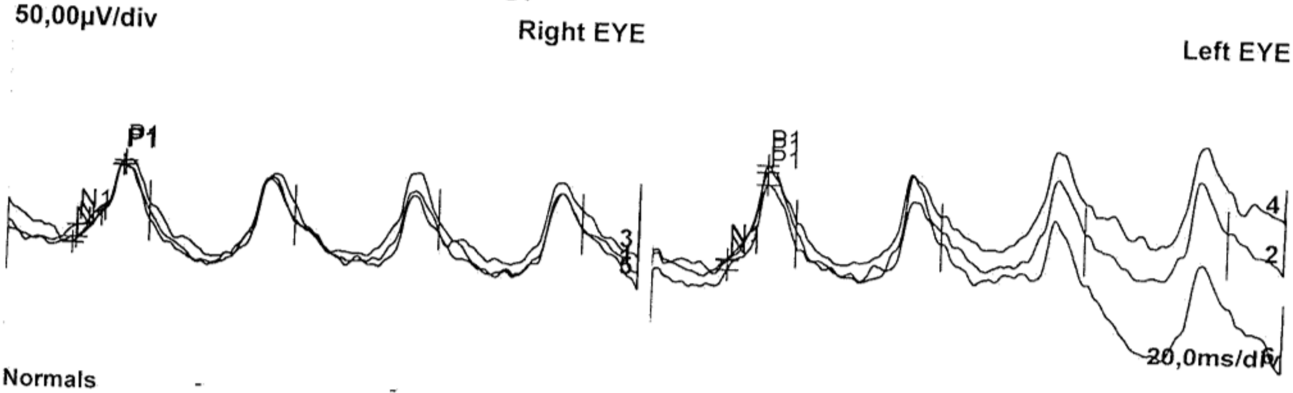

Normals - -

Channel	N1 [ms]	P1 [ms]	N1-P1
**1 R-1**	15,9	28,2	94,4µV
**3 R-1**	16,7	28,2	91,6µV
**5 R-1**	17,0	27,6	72,1µV
**2 L-2**	17,9	27,0	113µV
**4 L-2**	17,9	27,0	103µV
**6 L-2**	17,9	27,0	102µV
