# Supplementary material for: Multimodal Imaging of Dual BEST1/EFEMP1-Associated Hereditary Macular Disease
Source: J Clin Med. 2026 Jul 13;15(14):5495. doi: 10.3390/jcm15145495 (PMC13412455; doi:10.3390/jcm15145495)
Supplement: Supplementary file 1 [file jcm-15-05495-s001.zip › PERG_1 .pdf]

Diagnosis:

Pattern-ERG

5,00µV/div

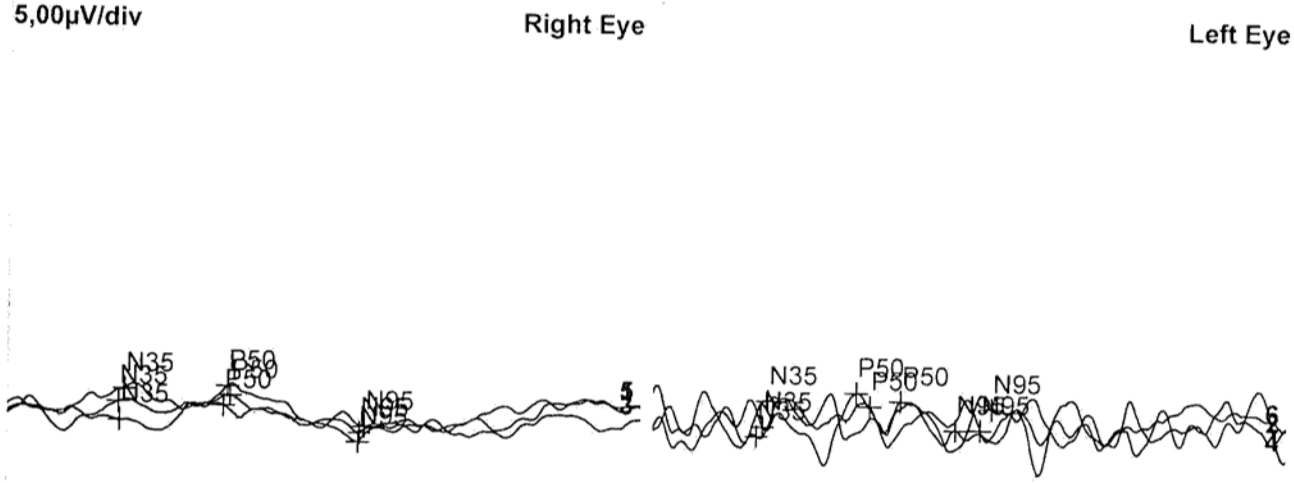

20,0ms/div

Normals	25-45	40-60	85-105		
**Channel**	**N35 [ms]**	**P50 [ms]**	**N95 [ms]**	**N35-P50**	**P50-N95**
**1 R-1 48 min**	32,1	62,0 (!)	101,8	127nV	940nV
**3 R-1 48 min**	33,8	63,1 (!)	100,0	202nV	2,65µV
**5 R-1 48 min**	32,4	63,8 (!)	100,4	1,28µV	1,75µV
**2 L-2 48 min**	32,8	70,5 (!)	96,2	35,6nV	330nV
**4 L-2 48 min**	29,6	61,6 (!)	93,0	1,53µV	1,11µV
**6 L-2 48 min**	31,4	57,8	85,9	1,71µV	1,77µV
