# Supplementary material for: Multimodal Imaging of Dual BEST1/EFEMP1-Associated Hereditary Macular Disease
Source: J Clin Med. 2026 Jul 13;15(14):5495. doi: 10.3390/jcm15145495 (PMC13412455; doi:10.3390/jcm15145495)
Supplement: Supplementary file 1 [file jcm-15-05495-s001.zip › PERG_2.pdf]

Diagnosis:

Pattern-ERG

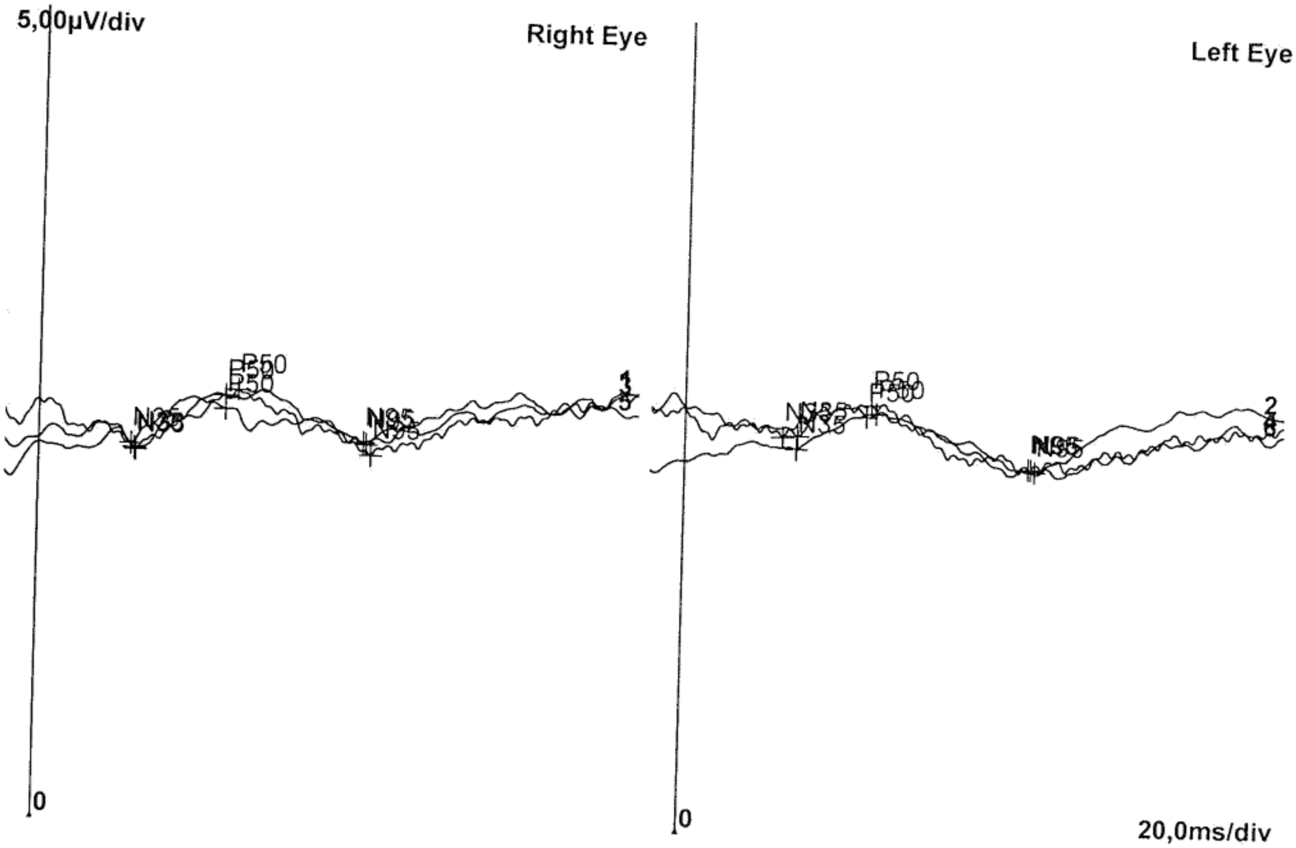

Normals Channel	N35 [ms]	P50 [ms]	N95 [ms]	N35-P50	P50-N95
1 R-1 48 min	27,5	53,2	92,3	2,69µV	2,19µV
3 R-1 48 min	27,8	56,7	94,4	3,09µV	3,11µV
5 R-1 48 min	26,4	53,2	93,0	1,80µV	1,60µV
2 L-2 48 min	31,7	54,2	99,3	1,83µV	2,70µV
4 L-2 48 min	27,8	52,8	98,3	1,70µV	3,02µV
6 L-2 48 min	32,1	51,4	97,6	1,01µV	2,39µV
